# Prevalence and correlates of forgone care among adult Israeli Jews: A survey conducted during the COVID-19 outbreak

**DOI:** 10.1371/journal.pone.0260399

**Published:** 2021-11-22

**Authors:** Perla Werner, Aviad Tur-Sinai

**Affiliations:** 1 Department of Community Mental Health, University of Haifa, Haifa, Israel; 2 Department of Health Systems Management, The Max Stern Yezreel Valley College, Yezreel Valley, Israel; 3 School of Nursing, University of Rochester Medical Center, Rochester, NY, United States of America; University of Malta Faculty of Health Sciences, MALTA

## Abstract

Efforts to control the spread of the novel Coronavirus (COVID-19) pandemic include drastic measures such as isolation, social distancing, and lockdown. These restrictions are accompanied by serious adverse consequences such as forgoing of healthcare. The study aimed to assess the prevalence and correlates of forgone care for a variety of healthcare services during a two-month COVID-19 lockdown, using Andersen’s Behavioral Model of Healthcare Utilization. A cross-sectional study using computerized phone interviews was conducted with 302 Israeli Jewish participants aged 40 and above. Almost half of the participants (49%) reported a delay in seeking help for at least one needed healthcare service during the COVID-19 lockdown period. Among the predisposing factors, we found that participants aged 60+, being more religious, and reporting higher levels of COVID-19 fear were more likely to report forgone care than younger, less religious and less concerned participants. Among need factors, a statistically significant association was found with a reported diagnosis of diabetes, with participants with the disease having a considerably higher likelihood of forgone care. The findings stress the importance of developing interventions aimed at mitigating the phenomenon of forgoing care while creating nonconventional ways of consuming healthcare services. In the short term, healthcare services need to adapt to the social distancing and isolation measures required to stanch the epidemic. In the long term, policymakers should consider alternative ways of delivering healthcare services to the public regularly and during crisis without losing sight of their budgetary consequences. They must recognize the possibility of having to align medical staff to the changing demand for healthcare services under conditions of health uncertainty.

## Introduction

The Coronavirus (COVID-19) pandemic confronts healthcare systems with unprecedented challenges. As of December 2020, with close to 74 million confirmed cases in 220 countries, a worldwide death toll of over 1,650,000 cases [1], and no foreseeable treatments or vaccines, countries around the world are relying on non-pharmacological public health measures to respond to the outbreak. These measures include isolation, social distancing, and quarantine [2].

While these measures were proven essential for controlling the spread of the pandemic, preventing excess demands on health care systems, and for limiting morbidity and associated mortality [3], they are accompanied by serious negative consequences. Of particular concern are the short- and long-term economic consequences [4], the adverse psychological and psychosocial effects [5, 6], and the undesirable impact on physical health caused mostly by forgone care.

Defined as delaying or relinquishing seeking for needed care [7], *forgone care* has been noted as one of the most worrisome consequences of the COVID-19 pandemic. With the majority of the resources directed towards the acute care of the pandemic, forgoing care for primary or specialty routine care has affected the quality of care, as well as of the physical and mental health of children, as well as adults [8–10]. Consequently, there is utmost importance to understand this phenomenon. However, except for an online poll conducted in April 2020 by the American College of Emergency Physicians, there is no empirical knowledge on the topic. This is surprising, as results of the above-mentioned survey showed that approximately one-third of the 2,201 adults participating in the study reported delaying or avoiding medical care in the last 30 days [11]. Despite the importance of this survey, it was not guided by any theoretical framework, and examined forgone care globally, without differentiating between different types of healthcare services. Our study intends to close these gaps in the field.

Thus, the aim of the present study was to assess the prevalence and correlates of forgone care during a two-month COVID-19 lockdown for a variety of healthcare services, using Andersen’s Behavioral Model of Healthcare Utilization [12]. This model posits that use of health services is a function of predisposing, enabling, and need factors. Predisposing factors reflect the propensity to use services and include demographic characteristics and health beliefs such as attitudes, values, and knowledge. Enabling or impeding factors reflect the resources and ability (or lack thereof) to find and access services. Health insurance is a traditional variable representing these factors. Finally, need factors reflect perceived needs for health care services such as specific health conditions. Although Andersen’s model has been applied in numerous studies focused in different diseases or settings, as stated by [13], "…it is near impossible to identify the factor having the "strongest influence" on health services utilization" (p. 14).

Our study was conducted among a sample of Israeli Jews and examined forgone for a variety of services covered by the basic version of the Israeli Health Insurance Law–called the healthcare "basket". Israel provides a good context for our study for several reasons. First, it provides a universal health insurance system. Under this law all citizens are entitled to receive "based on principles of justice, equality, and mutual assistance” (State Health Insurance Law 1994), a minimum packet of services (called the "basket of services"). The medical services basket includes the entire range of services, drugs, medical equipment and devices that the insured public has a right to receive, and which are provided by the HMOs at no charge, with the exception of services for which a copayment is required. The “basket of services” is being updated every year with new health technologies and medications, but due to budget limits not all healthcare services and medication are fully covered in it. Second, after the first cases of COVID-19 were identified, the Israeli government initiated measures to control the spread of the virus. These measures included enforcing social distancing, followed by the closure of public spaces, including schools, and putting an end to national and international travel. On March 19, 2020, the government declared a national state of emergency, and on April 14, 2020, a two-month partial lockdown was imposed [14].

## Methods

### Design and procedure

This cross-sectional study used computerized phone interviews to examine the prevalence and correlates of forgone care (including predisposing, enabling and need factors) for a variety of healthcare services during a two-month COVID-19 lockdown, with a sample of the adult (40+) Jewish population in Israel. This age group was selected because it is clear that older persons are at a higher risk of mortality as a result of COVID-19 than younger persons [15].

Phone calls were made using random digit dialing, with the adult having the most recent birthday designated as the respondent from all available respondents in each household. If that person was absent, then the next eligible adult was selected as the respondent. As stated above, the inclusion criterion was aged 40 and above. Exclusion criteria included: language difficulties, difficulties understanding the questions, and impossible to contact after three attempts. After providing verbal informed consent, participants were interviewed by trained interviewers.

Of the 604 phone numbers called, 297 individuals refused to participate–i.e., a 49.2% response rate. Reasons for refusal included not having time (n = 220) and language problems (n = 73). Additionally, four potential participants discontinued the interview, and five non-Jewish participants were removed from the analyses, leaving a final sample of 302 adults. Data collection was completed between May 15 and May 24, 2020

### Measures

#### Dependent variable—Forgone care

Participants were asked whether during the lockdown period they experienced a situation in which they had a need for healthcare, but did not seek/receive it. Forgone care was assessed for 13 healthcare services included in the Israeli "basket of services": family physician, expert physician, para-professional, hospital outpatient clinic, radiology, surgery, early-detection oncology tests, cardiac rehabilitation, nursing services, mental health services, child development care, geriatric services, and emergency department). Potential responses for each one of the services included: 1. "Didn’t have the need for this healthcare service"; 2. "Had the need and used the service in person"; 3. "Had the need and used the service via phone or video"; 4. "Had the need, but did not use it because the HMO cancelled the service"; and 5. "Had the need, but did not use the service".

#### Independent variables

According to Andersen’s model, these included predisposing, enabling/impeding, and need factors.

#### Predisposing factors

These included demographic characteristics and health beliefs.

Demographic information included: gender, age (continuously), number of years of education, marital status (married, living in couple, single, widowed), employment (employed, retired, unemployed), and religiosity. Religiosity included four categories—secular, traditional, religious, Orthodox, varying in their religious commitment, including the observance of beliefs and practices. For example, while no Orthodox or religious Jew will handle money or travel during the Sabbath, traditional Jews do so.

Health beliefs included perceived susceptibility and fear about contracting COVID-19:

*Perceived susceptibility* was assessed with a single question: “How likely do you think it is that you will contract COVID-19?” Answers were rated on a 5-point Likert-type scale, ranging from 1 = not at all likely to 5 = very likely.

*Fear* was assessed with a single question: “How much do you fear contracting COVID-19?” Answers were rated on a 5-point Likert-type scale, ranging from 1 = not at all worried to 5 = very worried.

#### Enabling factors

These factors included net monthly income (below the average, average, and above the average), and having private health insurance.

#### Need factors

These factors included reported diagnoses of heart disease, high blood pressure, respiratory disease, diabetes, and other chronic diseases.

### Ethical considerations

The study’s protocol was reviewed and approved by the research ethics committee of the University of Haifa, Israel (reference number 253/20). Since the study did not include the collection of any information or records that can be used to ascertain the identity of the participants by the researchers, and was conducted through telephone, we did not obtain a written consent. However, before starting the interview process, potential participants were read the aim of the study, its importance, the names and affiliation of the researchers, and an estimation of the time of the interview. Additionally, they were clarified about the process by which they were selected, about the anonymity of all data collection, and about their right to refuse or discontinue the interview at any time. Only after receiving the potential participants’ verbal consent, the interview process began.

### Statistical analyses

Descriptive statistics (means, standard deviations, percentages) were used to describe the sample and the main variables. The overall rate of forgone care was calculated for services reported as needed by at least 15% of the participants. These included visits to family physician, specialist physicians, para-professionals, and to a hospital outpatient clinic. We defined a medical service as forgone care if the participants reported they did not use the needed service at all (because of choice or unavailability of the needed service–scores 4 and 5 in the dependent variable). Multivariate logistic regression was used to estimate ORs of forgone care. All explanatory variables (i.e., predisposing, enabling, and need factors) were recoded as categorical variables. We assessed explanatory variables for the presence of significant multicollinearity and found none. All analyses were conducted using STATA, version 15.1 [16].

## Results

### Participants

As can be seen in Table 1, the majority of the participants were female (59.27%), secular (52.05%), and employed (55.09%), and reported having a below-average income (63.35%). Their mean age was 66 (S.D. 12.63; range 41–99 years), and they had an average of 14 years of education (S.D. 3.38; range 1–25 years). Thirty percent of the participants reported having a diagnosis of high blood pressure, and close to 20% reported a diagnosis of diabetes. Other diagnoses were uncommon in this sample and were not entered in further analyses. Regarding COVID-19, only 10% of the sample reported having had some symptoms related to the disease, but none of the participants were diagnosed with it.

**Table 1:** Participants’ characteristics (n = 302).

Characteristic	Percentage/mean (SD)
Gender (%)	
Male	40.73
Female	59.27
Mean (S.D.) age	65.94 ± 12.63
Mean (S.D.) number of years of education	14.43 ± 3.38
Religiosity (%)	
Secular	52.05
Traditional	26.03
Religious	11.64
Orthodox	10.27
Employment (%)	
Employed	55.09
Not employed	28.07
Retired	16.84
Net monthly income (%)	
Below average	63.35
Average	23.84
Above average	12.81
Reported diagnoses (%)	
Blood pressure	30.13
Diabetes	18.54
Heart disease	10.26
Pulmonary disease	5.30
Other chronic disease	9.6

Source: Authors’ analysis of data for May 2020 from a computerized phone interviews with a sample of the adult (40+) Jewish population in Israel.

### Prevalence of forgone care

As can be seen in Table 2, at least 15% of the participants reported having the need to visit their family physician, a specialist, a paraprofessional, or needing a consultation at an outpatient clinic. The percentage of participants reporting needing other medical services during the lockdown was lower (between 14% for the need of radiology services and less than 2% for geriatric services). Despite these low levels of need, between a quarter (for nursing services) and up to two-fifths (for surgery, cardiac rehabilitation, mental health services, and child development care) of the participants in need of these services reported delaying their use. However, as explained in the statistical analyses section, due to the small number of participants in need of these services, they were not included in the calculation of the overall prevalence of forgone care. Thus, as shown in Table 3, overall, almost half of the participants (49%) reported they had delayed seeking medical help for at least one of the four most needed types of medical services.

**Table 2:** Need and use of healthcare services in Israel’s basket of services (%) (n = 302).

Type of service	Service not needed	Service needed^a^			
		Used service in person	Used service via phone/video	Delayed using the service	HMO cancelled the use of service
Family physician	40.73	35.20	30.17	33.52	1.12
Expert physician	53.97	48.20	7.19	38.13	6.47
Para-professional^b^	84.44	48.94	6.38	34.04	10.64
Hospital outpatient clinic	79.47	48.39	6.45	37.10	8.06
Radiology	86.00	69.05		30.95	
Surgery	96.03	58.33		41.67	
Early-detection oncology tests	97.99	66.67		16.67	16.67
Cardiac rehabilitation	96.61	60.00		30.00	10.00
Nursing services	93.38	75.00		25.00	
Mental health services	96.69	60.00		40.00	
Child development care	97.68	57.14		42.86	
Geriatric services	98.34	60.00		40.00	
Emergency department	92.31	78.26		21.74	

Source: Authors’ analysis of data for May 2020 from a computerized phone interviews with a sample of the adult (40+) Jewish population in Israel.

Notes

^a^Percentages are calculated for those who reported needing the service for each type of medical service.

^b^Includes PT and OT services.

**Table 3:** Percentage of forgone care for the most needed medical services.

Type of service	Did not forgo care	Forgone care
Family physician (n = 179)	65.37	34.63
Expert physician (n = 139)	55.39	44.61
Para-professional (n = 47)	55.32	44.68
Outpatient hospital clinic (n = 62)	54.84	45.16
**At least one of these services was forgone (n = 221)**	**50.68**	**49.32**

Source: Authors’ analysis of data for May 2020 from a computerized phone interviews with a sample of the adult (40+) Jewish population in Israel.

### Correlates of forgone care

Table 4 displays the results of the multivariate logistic regression used to examine the determinants of forgone care. According to the Andersen’s model, the logistic regression included predisposing factors (demographic characteristics and health beliefs), enabling/impeding factors (income, and having private health insurance), and need factors (reported diagnoses). All examined variables were included in the regression, with the exception of reported diagnoses of heart disease, pulmonary disease, and other chronic disease, due to the small number of participants reporting these diagnoses. Odds ratio are presented in the table.

**Table 4:** Logistic regression model for forgone care (n = 188)^a^.

	OR (SE)	95% CI
Constant	0.085 (0.081)	[0.013, 0.549]
**Predisposing factors**		
Male	0.820 (0.292)	[0.408, 1.648]
Age (ref: age 40–49)		
50–59	2.691 (2.005)	[0.625, 11.593]
60–69	4.583** (3.112)	[1.211, 17.347]
70–79	6.262** (4.977)	[1.319, 29.736]
80+	7.376** (6.651)	[1.260, 43.189]
Married/living in a couple (ref: single/divorced/widowed)	1.213 (0.480)	[0.558, 2.635]
Religiosity (ref: secular)	1.899* (0.696)	[0.925, 3.896]
Education (ref: 12 years or less)	1.535 (0.567)	[0.744, 3.167]
Employment (ref: retired)		
Employed	1.147 (0.671)	[0.365, 3.607]
Unemployed	1.186 (0.694)	[0.377, 3.732]
Susceptibility to contract COVID-19	0.778 (0.143)	[0.543, 1.114]
Fear of contracting COVID-19	1.410** (0.195)	[1.075, 1.850]
**Enabling factors**		
Net monthly income (ref: below-average)		
Average	0.701 (0.267)	[0.332, 1.478]
Above-average	0.657 (0.374)	[0.215, 2.007]
Private health insurance	1.127 (0.405)	[0.557, 2.280]
**Need factors**		
High blood pressure	0.858 (0.326)	[0.407, 1.807]
Diabetes	2.735** (1.128)	[1.218, 6.140]
Log-likelihood	-114.25367	

Source: Authors’ analysis of data for May 2020 from a computerized phone interviews with a sample of the adult (40+) Jewish population in Israel.

Notes

^a^The number differed to that of Table 3, due to missing data.

* Significant at 10%

** significant at 5%

*** significant at 1%.

As can be observed, age, religiosity, and fear of contracting COVID-19 were the only predisposing factors significantly associated with forgone care. Specifically, participants aged 60 and above were more likely to report forgone care than younger participants (OR = 4.583, 95% CI of OR, 1.211–17.347, P = 0.025 for age group 60–69; OR = 6.262, 95% CI of OR, 1.319–29.736, P = 0.021 for age group 70–79; OR = 7.376, 95% CI of OR, 1.260–43.189, P = 0.027 for age group 80+), as were more religious ones (OR = 1.899, 95% CI of OR, 0.925–3.896, P = 0.080). Similarly, participants reporting high levels of fear of contracting the disease had a significantly higher likelihood of forgone care, compared to participants with lower levels of fear (OR = 1.410, 95% CI of OR, 1.075–1.850, P = 0.013).

No statistically significant associations were found for any of the enabling factors. Among need factors, a statistically significant association was found with a reported diagnosis of diabetes, with participants with the disease having a considerably higher likelihood of forgone care (OR = 2.735, 95% CI of OR, 1.218–6.140, P = 0.015).

## Discussion

COVID-19 has joined a list of pandemics affecting humankind across history. Although they might differ in their characteristics, they all share similar challenges and undesirable consequences [17]. Numerous publications have mentioned foregone or delayed care as one of the more damaging consequences of the spread of COVID-19. However, the vast majority were letters to the editor [8, 10], or other types of commentaries [9, 18, 19]. Understanding the scope of the problem and its correlates still requires a thorough, theory-driven empirical examination. This was the aim of the present study.

Our results showed that the greatest need for healthcare reported during the lockdown period was in relation to primary care services provided in the community, such as visiting the family physician, a specialist, a paraprofessional, or an outpatient clinic. This is worrisome since these services constitute the pillars of primary and secondary prevention. Thus, it is not surprising that healthcare systems worldwide are investing great efforts in adapting the delivery of primary care services to the new COVID-19 reality, including a shift to telephone and video consultations [20]. However, as can be seen in Table 2, these adaptations seemed to be more commonly used for family physicians (as close to one-third of the participants reported having a video or phone consultation with their physician), than for other types of primary care services.

Despite these adaptations, our study showed that one out of two Israelis (49%) aged 40 and over in our sample, reported delaying seeking help for at least one needed medical care service during the COVID-19 lockdown period. Studies assessing forgone care in non-epidemic situations reported lower percentages among samples of chronically ill, middle aged, and elderly patients [7, 21], and among younger samples [22]. In Israel, as well, a study assessing forgone care due to costs and not during the pandemic, showed that about ten percent of older adults reported relinquishing care [23]. This is not surprising since, as stated above, the fear accompanying COVID-19 outbreak, together with the strict measures adopted to prevent the transmission of the virus, and the heavy toll on health care providers, have resulted in the high prevalence of forgone care found in our study.

Using Andersen’s Behavioral Model as our conceptual framework, we found three predisposing factors (age, religiosity, and fear of contracting COVID-19) and one need factor (having a reported diagnosis of diabetes) to be significantly associated with forgone care. Contrary to previous studies [7, 21], we found that older age increased the chance of reporting forgone care. Two explanations might be provided for this discrepancy. First, while the above-mentioned studies were conducted among chronically ill adults, our sample included small percentages of participants reporting a chronic disease. Second, and most importantly, the present study assessed seeking medical help during the outbreak of a highly contagious disease affecting older persons more seriously than others [15], a fact that was constantly stated in the Israeli media, and which might have elicited higher levels of fear among this age group. Unfortunately, this manuscript does not examine differences between age groups. Future studies should do so in order to shed further light to the association between forgone care and age.

Fear of contracting COVID-19 was the second predisposing factor increasing the likelihood of forgone care. While an increasing amount of publications are focused on fear of contracting the disease as one of the most detrimental psychological consequences of the current pandemic [8, 24, 25], our study empirically confirms its impact on delaying help-seeking, which might translate into serious medical complications in the future. Finally, persons defining themselves as more religious also had a higher (although marginally significant) likelihood of delaying the use of medical services. This finding corroborates previous publications in a variety of areas which found that religious people are less inclined than secular people to seek medical help from professionals [26]. Moreover, this finding might also be the result of the much stricter measures enforced by the Israeli government in several religious towns and neighborhoods because of their very high contagion rates.

None of the enabling factors were significantly associated with forgone care. This is an interesting finding, hinting that, as will be discussed later, during epidemics, the decision to utilize or delay the use of a medical service relies less on economic factors, such as level of income or the availability of private insurance. Finally, having a reported diagnosis of diabetes (a need factor) considerably increased the likelihood of delaying medical care help seeking. Previous studies have reported similar associations between forgone care and having a chronic disease, including diabetes [27]. Since having a diagnosis of diabetes was demonstrated to considerably increase the risk of COVID-19 morbidity and mortality [28], media campaigns in Israel constantly stress persons with diabetes as a high-risk group. This might explain these individuals’ propensity to avoid seeking help, most probably because of their fears of contracting the disease.

Forgoing healthcare services under conditions of medical uncertainty may affect individual’s state of health in both the short and long terms. It may also have meaningful implications for persons employed in the healthcare system, because it is assumed that they should provide the public with healthcare services commensurate with demand. Therefore, if demand for these services declines under conditions of extreme stress, personnel supply may adjust itself to the new level of demand, personnel may become portable, and, in the extreme case, it may be necessary to cut back on supply of personnel either temporarily or on a longer-term basis.

The findings of this study emphasize that the public may change its patterns of consumption of healthcare services at times of lockdown. As a consequence of this conduct, the patterns of services offered by medical staff who are on the frontline of the healthcare services, must adapt to the changing demand for healthcare services under conditions of health uncertainty.

This study has several limitations. First, the sample included only Jewish participants. Future studies should examine prevalence and correlates of forgone care among Israeli Arabs. Second, our cross-sectional design does not allow for causal interpretations. Third, the use of self-reported measures might be associated with social desirability bias. However, confidentiality issues were explained to participants at the beginning of the interview, which we hope encouraged honest responses. Fourth, although our questionnaire included items examining the presence of a variety of chronic diseases’ diagnosis, the small percentages of participants reporting having a chronic disease other than diabetes, prevented us from examining associations with conditions such as heart problems, pulmonary diseases and other. Finally, although our results suggest the important role of fear in the decision to forgo care, we did not specifically explore other reasons. Future studies should address these limitations, as obtaining a more detailed and specific information on these topics might help develop interventions for reducing the prevalence of forgone care.

Despite these limitations, the present study has important conceptual and practical implications. Conceptually, it offers several ideas regarding the use of Andersen’s behavioral model as a reasonable framework for understanding the factors associated with forgone care during an epidemic outbreak. This model has been used extensively to explain the use of healthcare services. However, it is increasingly noted that the context of the studies–including the type of services and populations examined–affect the associations with the model’s main factors [13]. Thus, for example, in studies assessing primary care services, predisposing and enabling factors seem to be the most important correlates of use of services, while need factors are the most important ones for assessing emergency services [29]. In our study, model appropriateness was examined in regard to the forgoing of primary care medical services, but need factors were the stronger correlate. This might be the result of the stress and tension associated with the COVID-19 outbreak, leading older adults (who are constantly stated as being at high risk for the disease) to perceive seeking help for primary care services as an emergency situation. Future studies might want to further examine this possibility.

Practically, our study provides empirical evidence regarding the prevalence of forgone care during this ongoing epidemic. Undoubtedly, this is a topic requiring immediate attention by public health authorities, as it may lead to serious health, mental, and economic complications in the future. Older persons, those with increased levels of fear of contracting the disease, and those with a diagnosis of diabetes, might be unique targets for interventions aimed at reducing the extent of the phenomenon. Based on these findings, we recommend primary care professionals to outreach proactively these groups in order to try to prevent them from receiving adequate care. Finally, there is need to create and educate the public to use nonconventional ways of consuming healthcare services during times of crisis.

The COVID-19 pandemic has resulted in extraordinary challenges to healthcare systems. Our findings showed that forgone of health services is one of the most significant, complex, and serious ones, and will have substantial impact on future health outcomes. It is imperative therefore to take actions to mitigate these negative consequences. In the short term, healthcare services need to be adapted to the social distancing and isolation measures required to limit the epidemic. This includes adopting strict and evident hygienic and preventive measures, such as the use of masks and keeping with social distancing, in order to reduce the fear of older people to visit physically the clinics. Additionally, there is need to increase and optimize the provision of telecare services. This step is not restricted to the development and availability of these technologies, but also to increased efforts to educating and reassuring the public about the feasibility and safety of these methods, and examining the factors associated and differentiating between users and non-users of these new ways of delivering care. In the long term, policymakers and healthcare planers should consider alternative ways through which health services can be provided to residents on a regular basis, even on times of crisis, while considering their budgetary consequences.
